# Elevated Neurofilament Light Chain in Cerebrospinal Fluid and Plasma Reflect Inflammatory MRI Activity in Neurosarcoidosis

**DOI:** 10.3390/brainsci11020238

**Published:** 2021-02-14

**Authors:** Keld-Erik Byg, Helle H. Nielsen, Tobias Sejbaek, Jonna Skov Madsen, Dorte Aalund Olsen, Nina Nguyen, Astrid Kindt, Jakob Grauslund, Zsolt Illes, Torkell Ellingsen

**Affiliations:** 1 Department of Neurology, Odense University Hospital, J.B. Winsloewsvej 4, 5000 Odense, Denmark; Helle.Hvilsted.Nielsen@rsyd.dk (H.H.N.); Zsolt.illes@rsyd.dk (Z.I.); 2 BRIDGE—Brain Research—Inter-Disciplinary Guided Excellence, Department of Clinical Research, University of Southern Denmark, J.B. Winsloesvej 19, 5000 Odense, Denmark; Tobias.Sejbaek@rsyd.dk; 3 Rheumatology Research Unit, Odense University Hospital, J.B. Winsloewsvej 4, 5000 Odense, Denmark; torkell.ellingsen@rsyd.dk; 4 Department of Neurobiology Research, Institute of Molecular Medicine, University of Southern Denmark, J.B. Winsloesvej 21, 5000 Odense, Denmark; 5 Department of Neurology, South West Jutland University Hospital of Southern Denmark, Finsensgade 35, 6700 Esbjerg, Denmark; 6 Department of Regional Health Research, University of Southern Denmark, J.B. Winsloesvej 19, 5000 Odense, Denmark; Jonna.Skov.Madsen@rsyd.dk; 7 Department of Biochemistry and Immunology, Lillebaelt Hospital, University Hospital of Southern Denmark, Beriderbakken 4, 7100 Vejle, Denmark; Dorte.Aalund.Olsen@rsyd.dk; 8 Department of Radiology, Odense University Hospital, J.B. Winsloewsvej 4, 5000 Odense, Denmark; nina.nguyen@rsyd.dk; 9 Department of Ophthalmology, Odense University Hospital, J.B. Winsloewsvej 4, 5000 Odense, Denmark; Astrid.kindt92@gmail.com (A.K.); Jakob.Grauslund@rsyd.dk (J.G.); 10 Department of Clinical Research, University of Southern Denmark, J.B. Winsloesvej 19, 5000 Odense, Denmark

**Keywords:** neurofilament light chain, biomarker, neurosarcoidosis, cerebrospinal fluid, MRI

## Abstract

Background: Damage to axonal cells releases neurofilament light chain (NFL) into the cerebrospinal fluid and plasma. The objective of this study was to investigate NFL as a potential biomarker of disease activity in neurosarcoidosis. MRIs were graded according to enhancing lesions at different central nervous system (CNS) sites. Results: In cerebrospinal fluid, levels of NFL were higher in neurosarcoidosis patients (*n* = 20) median 2304 pg/mL (interquartile range (IQR) 630–19,612) compared to 426 pg/mL (IQR 261-571) in extra-neurologic sarcoidosis patients (*n* = 20) and 336 pg/mL (IQR 194–402) in healthy controls (*n* = 11) (*p* = 0.0002). In plasma, levels of NFL were higher in neurosarcoidosis patients median 28.2 pg/mL (IQR 11.5–49.3) compared to 6.2 pg/mL (IQR 4.3–8.2) in extra-neurologic sarcoidosis patients and 7.1 pg/mL (IQR 6.2–9.0) in healthy controls (*p* = 0.0001). Levels in both cerebrospinal fluid and plasma were higher in neurosarcoidosis patients with moderate/severe enhancement than patients with mild enhancement on MRI (*p* = 0.009 and *p* = 0.005, respectively). To distinguish neurosarcoidosis patients from extra-neurologic patients and healthy controls, a cut-off level of 630 pg/mL in cerebrospinal fluid had 94% specificity and 79% sensitivity, while a cut-off level of 11.4 pg/mL in plasma had 97% specificity and 75% sensitivity. Conclusions: NFL levels in cerebrospinal fluid and plasma are significantly higher in neurosarcoidosis patients compared to extra-neurologic patients and healthy controls, and the levels correlate to the extent of inflammation on MRI.

## 1. Introduction

Sarcoidosis is an autoimmune disease characterised by non-caseous granulomatous inflammation. The annual incidence in Denmark is seven cases per 10^5^ individuals [1]. Approximately 5% of sarcoidosis patients have neurosarcoidosis (NS) [2], which has a heterogeneous clinical presentation and commonly involves cranial neuropathy, central nervous system (CNS) mass lesions, meningeal infiltration, aseptic meningitis, or myelitis [3,4]. Diagnosing NS is challenging as clinical symptoms may not reflect disease activity. Magnetic resonance imaging (MRI) and cerebrospinal fluid (CSF) findings are frequently non-specific [4,5], and only a few biomarkers have been investigated so far [6,7,8].

Neurofilaments (NF) are highly specific, and major structural neuronal cytoskeletal proteins that consist of four NF subunits. One subunit is the neurofilament light chain (NFL), which is exclusively found in neuronal cells [9]. Damage or disruption of the axonal membrane releases NFL via the extracellular space into the CSF and in lower concentrations to the blood [10]. The NFL is non-specific as to the cause of the CNS impact but can be used to measure disease activity and prognosis in several neurological diseases [11], infections [12], and head trauma [13].

This current study aims to investigate the clinical potential of NFL as a biomarker for disease activity in NS. Our objectives were (i) to compare CSF and plasma NFL levels among NS patients, extra-neurologic sarcoidosis (ENS) patients, and healthy controls (HCs), (ii) to compare CSF and plasma NFL levels in relation to MRI activity in the CNS, and (iii) to identify cut-offs in CSF and plasma NFL concentration that differentiate NS patients from ENS patients and HC and to define their specificity and sensitivity.

## 2. Materials and Methods

### 2.1. Study Population

In this observational cross-sectional study, patients were consecutively recruited from the Department of Neurology and Rheumatology of Odense University Hospital between January 2016 and August 2020. The number of ENS participants was matched to the number of NS patients.

The inclusion criteria were patients aged 18 years or over, biopsy showing systemic granulomatous disease, and symptoms consistent with sarcoidosis in accordance with the American Thoracic Society and European Respiratory Society [14]. Patients were excluded if an alternative diagnosis could not be ruled out or if they were diagnosed with a polyneuropathy other than small-fibre neuropathy.

The patients were categorised as either NS patients or ENS patients. All NS patients fulfilled the criteria for highly probable NS of the World Association of Sarcoidosis and Other Granulomatous Diseases [15]. Inflammation was defined by MRI findings compatible with NS or CSF with leucocytosis over 5 × 10^3^ cells/mL, protein elevation over 0.5 g/L, or presence of oligoclonal bands (OCB).

Healthy controls without any signs of neurological or systemic inflammatory diseases were recruited by the Department of Neurology.

### 2.2. Data Collection

Age, sex, medication, and chest X-ray findings were registered at baseline.

If NS was suspected, the patient underwent MRI scans and CSF examination. The CSF was analysed for cells, protein, and OCB. For biobanking, CSF samples were centrifuged at 450 G for 20 min, and plasma obtained from EDTA anticoagulated whole blood was centrifuged at 2000 G for 10 min. Both CSF and plasma were aliquoted in Sarstedt polypropylene tubes and stored at −80° Celsius until analysis.

### 2.3. Data Validation

The same neuroradiologist evaluated all MRI images. The MRI images were acquired with a 3-Tesla MRI scanner. The MRI scanning protocols include T2-weighted turbo spin-echo images, T1-weighted 3-dimensional (3-D) turbo field echo images, T2-weighted 3-D Flair sequence image, Diffusion-weighted imaging, Susceptibility weighted imaging sequence and T1-weighted 3-D sequence image after intravenous contrast. The MRI was graded by the number of affected anatomical sites visualised by post-contrast enhanced lesions. Supratentorial meningeal enhancement, infratentorial meningeal enhancement, parenchymal enhancement, and enhancing lesions in the cervical spine, thoracic spine, lumbar spine, or cauda equina all give one point each [8]. A score of 0 points was graded no enhancement, 1–2 points were graded mild enhancement, and a score of 3–6 points was graded moderate/severe enhancement.

NFL in the CSF and plasma were analysed using a commercially available NF Kit (Quanterix©, Billerica, MA, USA) for the Single Molecule Array (Simoa^®^) HD-1 Analyser) according to the manufacturer’s procedure. In-house CSF and plasma pools were used as internal controls and included in each assay for evaluating assay performance. The total coefficient of variation was <12%.

### 2.4. Statistical Analysis

Continuous data were presented as the median and interquartile range (IQR). Kruskal–Wallis test was used for comparison between groups, and a Wilcoxon rank-sum test was used for pairwise comparisons.

Categorical data were reported as frequency and percentage and compared using Fisher’s exact test. Logistic regression and receiver operating characteristic (ROC) analysis were used to identify the cut-offs for CSF and plasma NFL concentration to differentiate NS patients from ENS patients and HC, and to define their specificity and sensitivity. All analyses were performed in Stata 16.1 (StataCorp LCC, College Station, TX, USA). A *p*-value below 0.05 was considered significant.

## 3. Results

### 3.1. Baseline Characteristics

We included 20 patients with NS, 20 patients with ENS, and 11 HC (Table 1). The median age of NS patients was higher (51.6 years) than that of ENS patients (44.5 years) and especially of HC (37.0 years). The duration of symptoms before inclusion in the study was shorter in NS patients (median eight months) than ENS patients (33 months). None of the patients had severe comorbidities. The proportion of females was highest in the HC group. In NS patients, the most common neurological symptom was headache (*n* = 12) followed by vertigo (*n* = 11) and tinnitus (*n* = 10). Furthermore, objective pathological neurologic findings were nearly exclusively seen in NS patients. The two patient groups had similar numbers of patients on immunosuppression and distribution of X-rays stage.

CSF was examined in all the NS patients and the HCs, and the seven ENS patients with neurological symptoms. In NS patients, the CSF was abnormal in 95% (*n* = 19) of patients, where 16 patients had CSF pleocytosis (median cell counts 22.5 × 10^3^ cells/mL; IQR 18.0–40.4), 17 patients had elevated protein levels (median 0.59 g/L; IQR 0.55–0.99), and OCB was present in six patients.

Among NS patients, 60% (*n* = 12) had abnormal findings on MRI, of which nine had abnormalities of the cerebrum, and ten had abnormalities of the medulla spinalis.

### 3.2. Neurofilament Light Chain

**Figure 1:**
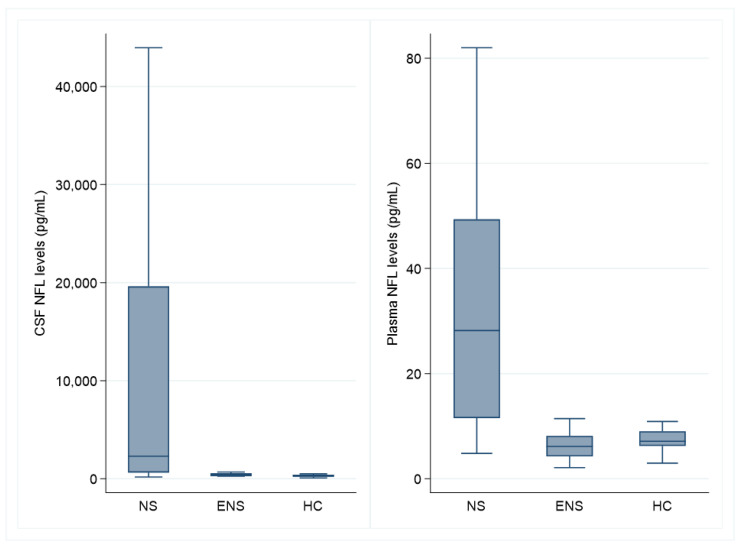
Box plot (Tukey method, without outliners) of neurofilament light chain levels in cerebrospinal fluid and plasma. NFL: neurofilament light chain, CSF: cerebrospinal fluid, NS: neurosarcoidosis patients (*n* = 20), ENS: extra-neurologic sarcoidosis patients (*n* = 7 in CSF and *n* = 20 in plasma), HC: healthy controls (*n* = 11).

NFL levels in the CSF were higher (*p* = 0.0002) in NS patients compared to ENS patients and HC (Figure 1). The median NFL level in the CSF was 2304 pg/mL (IQR 630–19,612) in NS patients, 426 pg/mL (IQR 261–571) in ENS patients, and 336 pg/mL (IQR 194–402) in HCs. Plasma NFL levels were also higher (*p* = 0.0001) in NS patients compared to ENS patients and HCs. The median plasma NFL was 28.2 pg/mL (QR 11.5–49.3) in NS patients, 6.2 pg/mL (IQR 4.3-8.2) in ENS patients, and 7.1 pg/mL (IQR 6.2–9.0) in HCs.

In both CSF and plasma, immunosuppression treatment on time of inclusion did not influence the NFL level in either NS patients or ENS patients. The CSF NFL level was median 3291 pg/mL (IQR 478–18,066) in NS patients on immunosuppression and 2304 pg/mL (IQR 630–19,612) in NS patients without immunosuppression (non-significant). The plasma NFL level was median 30.4 pg/mL (IQR 10.8-60.3) in NS patients on immunosuppression and 28.2 pg/mL (IQR 11.5–49.3) in NS patients without immunosuppression (non-significant).

In NS patients, we examined contrast-enhancing lesions on MRI (Table 2). Eight patients had no enhancement, five patients had mild enhancement, and seven patients had moderate/severe enhancement. There were significant differences between these three groups in both CSF (*p* = 0.004) and plasma (*p* = 0.003). Pairwise comparisons revealed a significant difference between NS patients with moderate/severe enhancement and those with mild enhancement, but not between mild and no enhancement. In CSF, the median NFL in moderate/severe enhancement was 28,977 pg/mL (IQR 19,612–30,239) compared to 689 pg/mL (514–2070) in mild enhancement (*p* = 0.009). In plasma, the median NFL in moderate/severe enhancement was 62.7 pg/mL (IQR 47.2–82.0) compared to 16.8 (16.0–16.8) in mild enhancement (*p* = 0.005).

**Figure 2:**
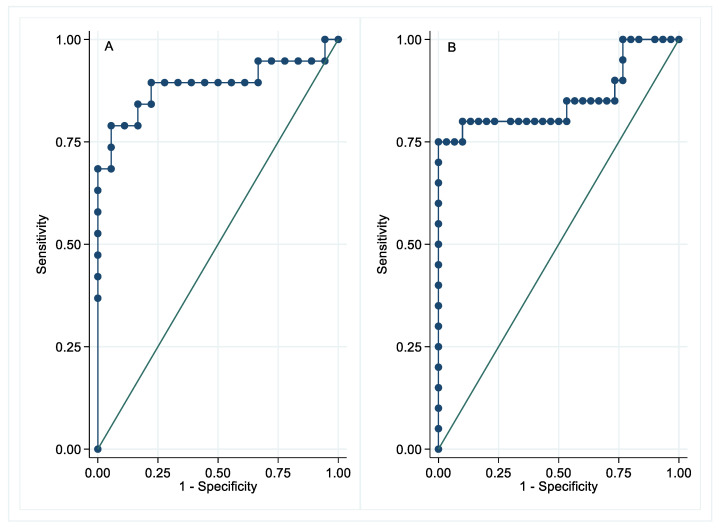
Receiver operating characteristic (ROC) curves of neurofilament light chain (NFL). (**A**) ROC curve of cerebrospinal fluid NFL levels between neurosarcoidosis (NS) patients versus extra-neurologic sarcoidosis (ENS) patients and healthy controls (HCs). The area under curve 0.8889, *p* = 0.05. (**B**) ROC curve of plasma NFL levels between NS patients versus ENS patients and HC. The area under curve 0.8550, *p* = 0.009.

Receiver operating characteristic (ROC) analysis (Figure 2) was performed on CSF and plasma NFL to differentiate NS patients from ENS patients and HCs. In CSF, the area under the ROC curve was 0.8889 and nearly reached significance (*p* = 0.05). A cut-off concentration of 630 pg/mL in CSF showed 94% specificity and 79% sensitivity. In plasma, the area under the ROC curve was 0.8550 and was significant (*p* = 0.009). A cut-off concentration of 11.4 pg/mL in plasma showed 97% specificity and 75% sensitivity.

## 4. Discussion

In this observational cross-sectional study, we investigated the NFL in CSF and plasma as a potential biomarker in NS. We found that NFL levels in both the CSF and plasma were higher in NS patients compared to levels in ENS patients and HC. The majority (75–80%) of NS patients had NFL levels in both CSF and plasma above our cut-off limit, in contrast to only one among ENS patients and none among HC. Furthermore, the NFL levels reflected the amount of inflammation visualised by MRI.

Different biomarkers have been studied in NS, e.g., angiotensin-converting enzyme [16,17], soluble interleukin-2 receptor (sIL-2R) [7,18], interleukin-6 (IL-6), interleukin-10 [6], and S100B [8,19]. sIL-2R, IL-6, and S100B have all been shown to be elevated in NS, but sIL-2R and IL-6 are non-specific, and S100B is not exclusively associated with neural cells [19]. NFL has the advantage of being specific for axonal damage but non-specific when it comes to aetiology [9]. Our study showed elevated CSF and plasma NFL in NS patients as an indication of axonal damage or disruption, in comparison to ENS patients and HC. NFL is often found to correlate with disease activity in various diseases causing neuroaxonal destruction [11], including autoimmune diseases. In systemic lupus erythematosus, increasing NFL concentrations have been associated with impaired psychomotor speed and motor function [20]. In primary Sjögrens syndrome and neuromyelitis optic spectrum disorders, the NFL concentrations have been associated with impaired motor function [20], and increasing disability [21]. In multiple sclerosis (MS) patients, NFL levels have been shown to correlate with future brain and cervical spinal volume loss [22]. Furthermore, a gradually increased NFL level relative to the number of enhancing lesions on MRI has been observed [23]. In line with this, we found a considerable elevation in both CSF and plasma NFL in NS patients with moderate/severe enhancement on MRI compared to patients with no or mild MRI enhancement. NFL is, therefore, a potential biomarker that could be useful in the management of NS and supplementary to MRI.

We used ROC analysis to establish a cut-off NFL concentration in CSF and plasma that could differentiate between NS patients and the two other groups. Although the inflammatory processes are different in MS patients, the cut-off limits were nearly the same [24], and the limits showed a good ability to distinguish NS patients from ENS patients and HC.

The limitations of this study were the small number of patients due to the low incidence of NS, even in a highly specialised university clinic. The three groups had different sex and age distributions. NFL is not influenced by sex [23], but older age is associated with increased levels of NFL, although this is mostly in patients over 55 years of age. The median age in all three groups was below this age, and it is, therefore, unlikely that age would have affected the NFL levels in this study [25]. Since CSF examinations were only performed in patients with neurological symptoms, CSF was examined in seven out of 20 ENS patients, which may weaken the statistical analyses.

## 5. Conclusions

This study has demonstrated that NFL is a potential biomarker in neurosarcoidosis that can help distinguish these patients from extra-neurologic sarcoidosis patients and healthy controls. In addition, the NFL level in both CSF and plasma reflected the inflammatory activity on MRI. Further extensive studies are required to evaluate NFL as a screening biomarker for neurosarcoidosis and its potential as a biomarker for disease activity and prognosis.

## Figures and Tables

**Table 1:** Baseline characteristics in patients with neurosarcoidosis or extra-neurologic sarcoidosis, and healthy controls.

	Neurosarcoidosis	Extra-Neurologic Sarcoidosis	Healthy Controls
*n*	20	20	11
Age, years (median, IQR)	51.6 (43.0–56.4)	44.5 (40.2–54.6)	37.0 (25.8–44.5)
Female (%)	9 (45)	14 (70)	10 (91)
Duration of symptoms, months (median, IQR)	8 (4–16)	33 (13–45)	
Neurological symptoms			
Headache (%)	12 (60)	6 (30)	
Tinnitus (%)	10 (50)	2 (10)	
Vertigo (%)	11 (55)	1 (5)	
Cranial nervous affection (%)	6 (30)	1 (5)	
Peripheral motor symptoms (%)	8 (40)	0	
Peripheral sensory symptoms (%)	10 (50)	0	
Chest X-ray (%)			
Stage 0	8 (40)	4 (20)	
Stage 1	9 (45)	12 (60)	
Stage 2	3 (15)	3 (15)	
Stage 3–4	0	1 (5)	
Immunosuppression (%)			
Glycocorticosteroid	4 (20)	5 (25)	
Methotrexate/Azatrioprin	2 (10)	5 (25)	

Values are median, interquartile range (IQR) or count (percentage). Chest X-ray Stage 0: normal chest x-ray, Stage 1: bilateral hilar lymphadenopathy, Stage 2: bilateral hilar lymphadenopathy and pulmonary infiltrations, Stage 3: pulmonary infiltrations without bilateral hilar lymphadenopathy, Stage 4: pulmonary fibrosis.

**Table 2:** NFL levels in CSF and plasma related to contrast-enhancing lesions on MRI.

	No Enhancement	Mild Enhancement	Moderate/Severe Enhancement	*p*-Value ^a^
*n*	8	5	7	
NFL in CSF (median, IQR)	820 (449–2635)	689 (514–2070)	28977 (19,612–30,239)	0.004
NFL in plasma (median, IQR)	9.5 (5.2–28.9)	16.8 (16.0–16.8)	62.7 (47.2–82.0)	0.003

Values are median, interquartile range (IQR) or count (percentage). NFL: neurofilament light chain. CSF: cerebrospinal fluid. No enhancement: no contrast enhancement. Mild enhancement: contrast enhancement in one or two anatomical sites. Moderate/severe enhancement: contrast enhancement in three to six anatomical sites. ^a^ Kruskal–Wallis test.

## Data Availability

The data underlying this article will be shared on reasonable request to the corresponding author.
